# Supplementary material for: A Likelihood Approach for Real-Time Calibration of Stochastic Compartmental Epidemic Models
Source: PLoS Comput Biol. 2017 Jan 17;13(1):e1005257. doi: 10.1371/journal.pcbi.1005257 (PMC5240920; doi:10.1371/journal.pcbi.1005257)
Supplement: S1 File — (TAR.GZ) [file pcbi.1005257.s014.tar.gz › HSPH_Online-SI-Revision/IPoi09/n10-extreme/n10-extreme_BENCH_summary.pdf]

peak -8	R0	$1/\theta_2$	inf	inflog	newly	newlylog	Reff	AttackRate
mean	22.4	35.5	133.6	0.6	354.4	0.7	21.4	22.8
median	23.7	34.	79.3	0.5	118.2	0.6	21.4	15.2
Quantile .1	11.8	17.1	60.2	0.4	63.6	0.4	11.4	6.3
Quantile .9	30.2	62.9	219.4	0.8	470.	0.9	32.4	46.6
30	36	31						
length	50							

peak -4	R0	$1/\theta_2$	inf	inflog	newly	newlylog	Reff	AttackRate
mean	12.2	25.2	65.9	0.3	64.	0.3	20.9	5.3
median	10.1	20.	59.5	0.3	51.3	0.3	19.7	3.9
Quantile .1	4.	6.6	42.5	0.2	36.2	0.2	9.4	1.2
Quantile .9	20.7	53.2	95.5	0.4	92.8	0.4	33.5	10.8
25	13	29						
length	50							

peak	R0	$1/\theta_2$	inf	inflog	newly	newlylog	Reff	AttackRate
mean	17.5	20.6	30.6	0.1	21.8	0.1	45.3	6.2
median	12.6	12.	20.3	0.1	18.8	0.1	43.8	3.5
Quantile .1	0.9	2.2	14.2	0.1	14.3	0.1	38.1	0.4
Quantile .9	37.2	57.3	52.1	0.3	32.8	0.2	53.4	12.7
1	0	7						
length	50							

peak +4	R0	$1/\theta_2$	inf	inflog	newly	newlylog	Reff	AttackRate
mean	18.1	22.9	66.3	0.2	48.3	0.2	35.3	6.3
median	14.	12.8	47.7	0.2	42.6	0.2	35.	3.5
Quantile .1	0.9	2.9	33.7	0.1	35.2	0.1	18.2	0.5
Quantile .9	39.1	58.7	108.9	0.4	66.1	0.3	46.8	13.3
0	0	8						
length	50							

peak +8	R0	$1/\theta_2$	inf	inflog	newly	newlylog	Reff	AttackRate
mean	18.	23.	124.7	0.3	92.1	0.3	26.7	6.2
median	14.	12.6	74.8	0.2	67.4	0.2	24.2	3.5
Quantile .1	1.8	3.1	48.7	0.2	48.9	0.2	9.3	0.5
Quantile .9	39.1	58.9	245.2	0.5	148.6	0.4	46.5	13.3
0	0	7						
length	50							
